# Supplementary material for: Differential Roles of Dendritic Cells in Expanding CD4 T Cells in Sepsis
Source: Biomedicines. 2019 Jul 18;7(3):52. doi: 10.3390/biomedicines7030052 (PMC6783955; doi:10.3390/biomedicines7030052)
Supplement: Supplementary file 1 [file biomedicines-07-00052-s001.pdf]

Supplemental Figure S1

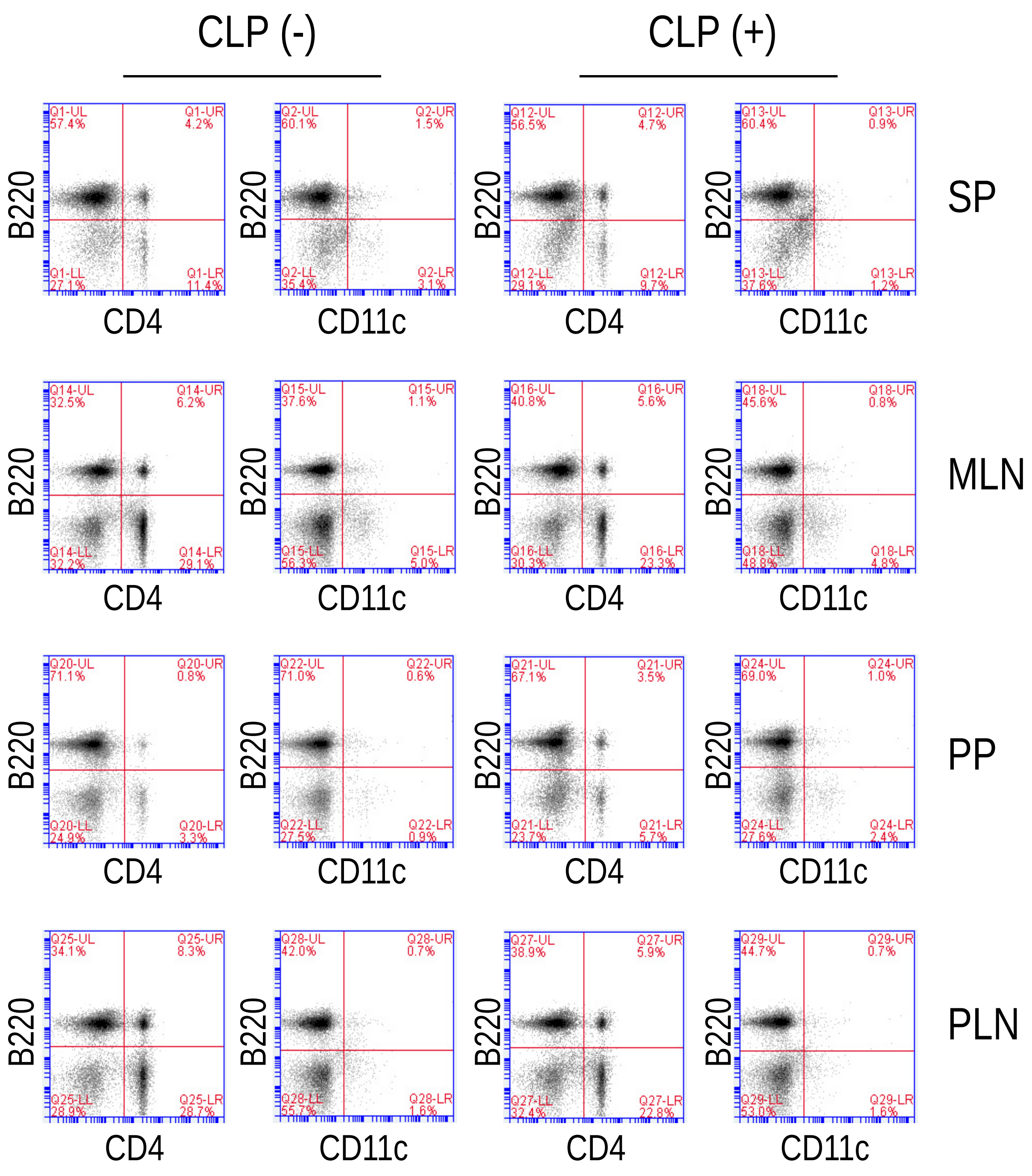

Supplemental Figure S1. The representative dot-plots are shown for sepsis-induced changes in ratio of $CD4^+$ T cells, $CD11c^+$ DCs, and $B220^+$ B cells in four different tissues (SP, MLN, PP, and PLN). For the PLN, inguinal LNs were isolated and used in this assay. CLP (-) and CLP (+) indicate control steady state and CLP-induced sepsis mice, respectively. The single-cell suspensions prepared from the tissues of three mice per group were subjected to antibody staining and flow-cytometry analysis. Data are representative of at least three independent experiments that show similar results.

Supplemental Figure S2

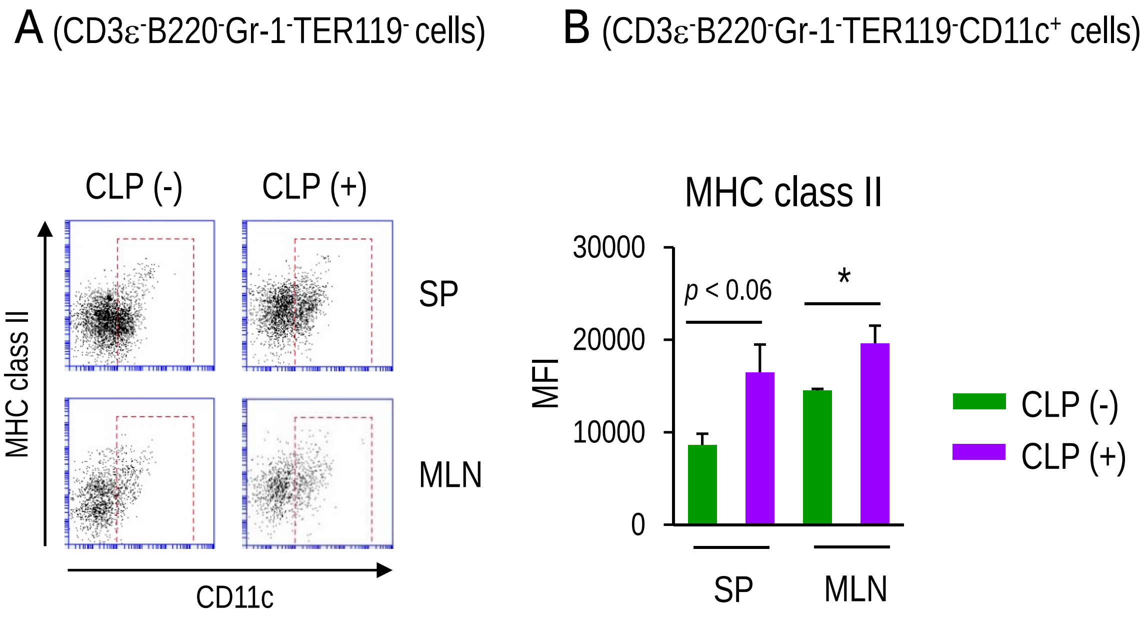

Supplemental Figure S2. Sepsis-induced change in expression of MHC class II on CD3ε$^-$B220$^-$Gr-1$^-$TER119$^-$CD11c$^+$ DCs. (**A**) Dot plots indicate the representative results of flow cytometry in which CD3ε$^-$B220$^-$Gr-1$^-$TER119$^-$ cells stained with mAbs to CD11c and MHC class II were analyzed. (**B**) The bar graph represents the mean ± SEM for MFI obtained from four different mice, in which green and purple bars indicate without (-) and with (+) CLP, respectively. MFI, mean fluorescent intensity. *$0.01 < p < 0.05$.

Supplemental Figure S3

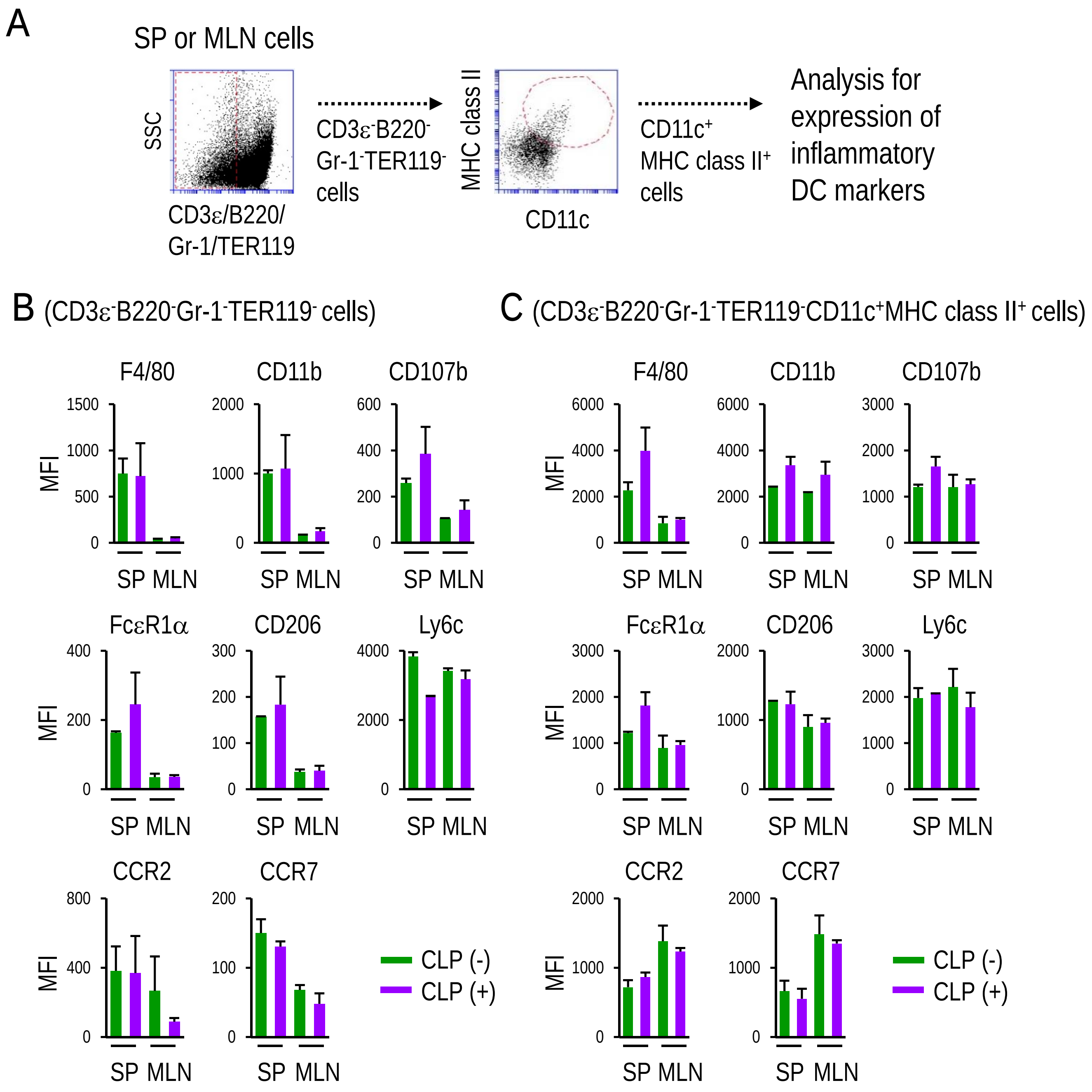

Supplemental Figure S3. Sepsis-induced change in expression of inflammatory DC markers. (**A**) The way of gating the cells for analyzing expression in inflammatory DC markers is shown. After staining total mononuclear cells from SP or MLN with mAbs to CD3ε, B220, Gr-1, TER119, CD11c, and MHC class II, the cells double-positive for CD11c and MHC class II were gated from CD3ε$^-$B220$^-$Gr-1$^-$TER119$^-$ cells for further analysis. CD3ε$^-$B220$^-$Gr-1$^-$TER119$^-$ cells (**B**) and CD3ε$^-$B220$^-$Gr-1$^-$TER119$^-$CD11c$^+$MHC class II$^+$ cells (**C**) were analyzed for their expressions of the markers (F4/80, CD11b, CD107b, FcεR1α, CD206, or Ly6c) and chemokine receptors (CCR2 and CCR7). The single-cell suspensions prepared from combined tissues of three mice per group were subjected to antibody staining and flow-cytometry analysis. Bar graphs represent the mean ± SEM obtained from two to three independent experiments.

Supplemental Figure S4

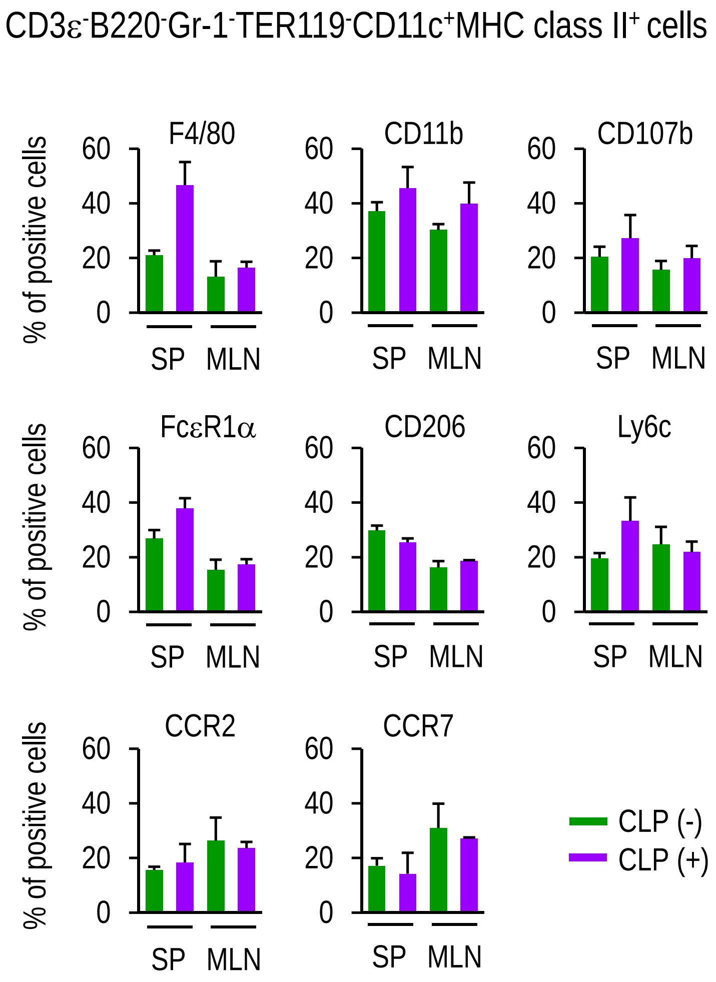

Supplemental Figure S4. Sepsis-induced change in ratio of inflammatory DC-marker expressing cells. Ratio of the cells that express the markers (F4/80, CD11b, CD107b, FcεR1α, CD206, or Ly6c) and chemokine receptors (CCR2 and CCR7) was measured to total CD3ε$^-$B220$^-$Gr-1$^-$TER119$^-$CD11c$^+$MHC class II$^+$ cells. The single-cell suspensions prepared from combined tissues of three mice per group were subjected to antibody staining and flow-cytometry analysis. Bar graphs represent the mean ± SEM obtained from two to three independent experiments.

Supplemental Figure S5

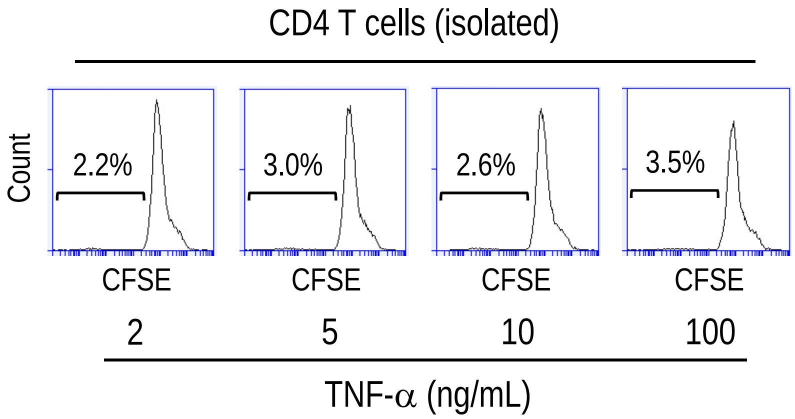

Supplemental Figure S5. TNF-α has no effect on CD4 T-cell proliferation. The CD4 T cells were isolated from single-cell suspensions of SP of Balb/c mice, fluorescently labeled with CFSE, treated with recombinant mouse TNF-α at the indicated concentrations, and further incubated for 3 days. Flow-cytometry histograms show representative results. The proliferation ratios were determined via measuring diluted fluorescence intensity of a histogram in flow cytometry in which the numbers inside squares represent the percentages of bracketed regions. Data are representative of at least three separate experiments.

Supplemental Figure S6

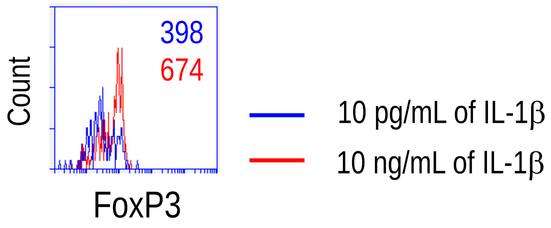

Supplemental Figure S6. The CD4 T cells proliferated or skewed by IL-1β display a property of regulatory T cells expressing FoxP3. The CD4 T cells stained with CFSE were cultured with either 10 pg/mL or 10 ng/mL of IL-1β for 3 days and analyzed for FoxP3 expression in CFSE-diluted cells. Flow cytometry histogram shows change in expression of FoxP3 in response to treatment with low (10 pg/mL, blue line) or high (10 ng/mL, red line) doses of IL-1β. The numbers inside square represent MFI for FoxP3 expression. MFI, mean fluorescent intensity. Data are representative of at least three independent experiments.
